# Oral nutritional supplement helps to improve nutritional status of dialysis dependent patients: a systematic review and meta-analysis of randomized controlled trials

**DOI:** 10.3389/fnut.2023.1294064

**Published:** 2023-11-23

**Authors:** Song Ren, Xiaoxiu Yao, Shangqing Ren, Yunlin Feng

**Affiliations:** ^1^Nephrology Department, Sichuan Provincial People’s Hospital, Chengdu, Sichuan, China; ^2^Department of Health Management, Damian Honghe Community Health Service Center of Longquanyi District, Chengdu, China; ^3^Robotic Minimally Invasive Surgery Center, Sichuan Provincial People’s Hospital, University of Electronic Science and Technology of China, Chengdu, China

**Keywords:** oral nutritional supplement, dialysis, nutritional status, systematic review, meta-analysis

## Abstract

**Background:**

The prevention and treatment of malnutrition holds remarkable implications in the overall management of dialysis patients. However, there remains a dearth of comprehensive evaluations regarding the impact of oral nutrition supplement (ONS) on all pertinent dimensions of malnutrition in the dialysis population.

**Methods:**

A systematic search was conducted in MEDLINE, EMBASE, and Cochrane Central Library. RCTs that had assessed the effects of oral nutritional supplement in dialysis-dependent populations were considered eligible. Outcomes included laboratory indicators, anthropometric measures, nutritional indices, dialysis adequacy, body composition analysis measures, and systemic inflammation indicators. The risk of bias was assessed according to Cochrane guidelines. Weighted mean difference (WMD) or standardized mean difference (SMD) with 95% confidence intervals (CIs) were pooled using a random-effects model.

**Results:**

In all, 22 RCTs with 1,281 patients were included. The pooled analyses revealed the serum ALB, BMI, nPCR, and MIS improved by 1.44 g/L (95% CI: 0.76, 2.57), 0.35 kg/m^2^ (95% CI: 0.17, 0.52), 0.07 g/(kg d) (95% CI, 0.05, 0.10), and −2.75 (95% CI, −3.95, −1.54), respectively following ONS treatments when compared to control treatments. However, no significant differences were observed in relation to the other outcomes examined. 15 studies were rated as having high risk of bias. Visual inspection of the funnel plot and Egger test argued against the presence of publication bias.

**Conclusion:**

ONS treatments helps to improve the nutritional status of dialysis dependent patients. More evidence is needed from future investigations with longer study duration and standardized procedures to support long-term use of ONS in this population.

**Systematic review registration:**

https://www.crd.york.ac.uk/prospero/, Identifier CRD 42023441987.

## Introduction

Malnutrition is highly prevalent among dialysis dependent patients (1, 2). The nutrients deficiency arises from a confluence of factors, namely reduced absorption of nutrients due to symptoms associated with renal failure, such as gastrointestinal discomfort, diminished appetite, acidosis, and depression, as well as the loss of nutrients during dialysis sessions, including proteins, glucose, and amino acids (3). Furthermore, malnutrition in renal failure patients can also be attributed to protein energy wasting (PEW), which is characterized by aberrant protein metabolism, progressive loss of skeletal muscle mass, low levels of serum albumin, and microinflammatory status (4).

Malnutrition significantly exacerbates the unfavorable prognosis of dialysis patients by interacting with the microinflammatory status, leading to accelerated development arterial disease which are closely associated with cardiovascular mortality (5). The compromised nutritional status of these patients places them at a heightened susceptibility to infectious disease and the subsequent catastrophic outcomes following severe infections. In addition, malnutrition complicates the correction of mineral and bone metabolism disorders. Therefore, the prevention and treatment of malnutrition holds remarkable implications in the overall management of dialysis patients (2).

Nutrition interventions have been demonstrated to be effective in improving the condition of dialysis patients (6). Among the various interventions, oral nutrition supplement (ONS) is considered an important strategy (6, 7). The commercially available ONS agents are not only convenient to consume, but have also been shown to effectively address malnutrition in dialysis patient without causing electrolytes disturbances, including calcium, phosphorus, and potassium (8). However, there remains a dearth of comprehensive evaluations regarding the impact of ONS on all pertinent dimensions of malnutrition in the dialysis population.

Therefore, we conducted this systematic review and meta-analysis to comprehensively synthesize the existing evidence on the use of ONS in dialysis dependent patients. The primary objective was to exam a wide range of outcomes pertaining to malnutrition in dialysis population, while prioritizing randomized clinical trials to ensure the highest level of evidence.

## Materials and methods

### Data sources and searches

A systematic search was conducted for eligible studies published up to July 17th, 2023 in EMBASE via Ovid, MEDLINE via PubMed, and Cochrane Central Library via Ovid according to the PRISMA (Preferred Reporting Items for Systematic Review and Meta-Analyses) statement (9). The search terms used text words relevant to chronic kidney disease, randomized clinical trial, and oral nutritional supplement (Supplementary Table S1). The study had been registered on PROSPERO (Identifier# CRD 42023441987).

### Study selection

Randomized clinical trials (RCTs) that had assessed the effects of oral nutritional supplement in dialysis-dependent populations were considered eligible for this study. There was no restriction on the nutritional agents in the supplement treatment or dialysis strategies.

The screening was conducted by two reviewers (SR and XY) independently following a standardized approach. The titles and abstracts of all returned records from database searching were carefully reviewed. Duplicates, non-original studies (e.g., reviews, editorials, commentaries, guidelines, proceedings, and secondary or subgroup analyses of RCTs), study protocols, case reports, animal studies, studies irrelevant to nutritional supplement, and studies in non-dialysis populations were excluded. Reference lists from the resulting articles after full text review were manually scanned to identify any relevant studies. Any discrepancy was resolved by a third reviewer (YF).

### Outcomes

This systematic review considered different aspects that could reflect the effects of nutritional supplement in dialysis-dependent patients. Briefly, these outcomes reflecting nutrition status were classified into the following six types: (1) laboratory indicators, including hemoglobulin, albumin (ALB), pre-albumin (pre-ALB), serum potassium, calcium, phosphorus, and lipid; (2) anthropometric measures, including body mass index (BMI), mid arm circumference (MAC), and mid arm muscle circumference (MAMC); (3) body composition analysis measures, including fat mass and lean mass; (4) nutritional indices, including normalized protein catabolic rate (nPCR) and malnutrition inflammation score (MIS); (5) dialysis adequacy evaluation reflected by Kt/V; and (6) systemic inflammation indicators, including C-reaction protein (CRP) and interleukin-6 (IL-6).

### Data extraction and quality assessment

Data from eligible studies were extracted by two reviewers (SR and XY) independently and compiled after cross-check. Any discrepancy was resolved by the third reviewer (YF). The extracted data included names of the first author, year of publication, geographical origin, number of patients in the overall study population, numbers of patients in the ONS and control groups, details of the ONS interventions, details of the control treatments, and details of reported outcomes. Information about potential sources of heterogeneity, such as the study intervals and sex makeup of the study population, was also collected for subgroup analysis.

### Critical appraisal of included studies

The risk of bias was independently assessed by two reviewers (SR and YF) based on the “Cochrane Handbook for Systematic Reviews of Interventions” imbedded in analysis software (10).

### Data synthesis and analysis

Data analysis and synthesis were conducted using Stata, version 17.0 (Stata) and RevMan, version 5.2 (RevMan). All studied outcomes were continuous variables. Means and standard deviations of changes from baseline were extracted (if reported in the original study) or calculated by subtracting the baseline values from the values after treatments based on a published equation (8). Weighted mean difference (WMD) or standardized mean difference (SMD) with 95% confidence intervals (CIs) were pooled using a random-effects model. Statistical heterogeneity was estimated using the *I*^2^ statistic (11). In studies that reported more than one invention of ONS, each intervention was treated as one independent interventional group and compared with the control treatment. The pooled results of individual outcomes were deemed having low, moderate, and high statistical heterogeneity if *I*^2^ was <25%, between 26% and 75%, and >75%, respectively (11). Subgroup analyses were also conducted based on the follow-up duration and dialysis mode. Publication bias was assessed using funnel plot analysis and Egger test. *p*-value of <0.05 was considered statistically significant.

## Results

### Search findings

**Figure 1:**
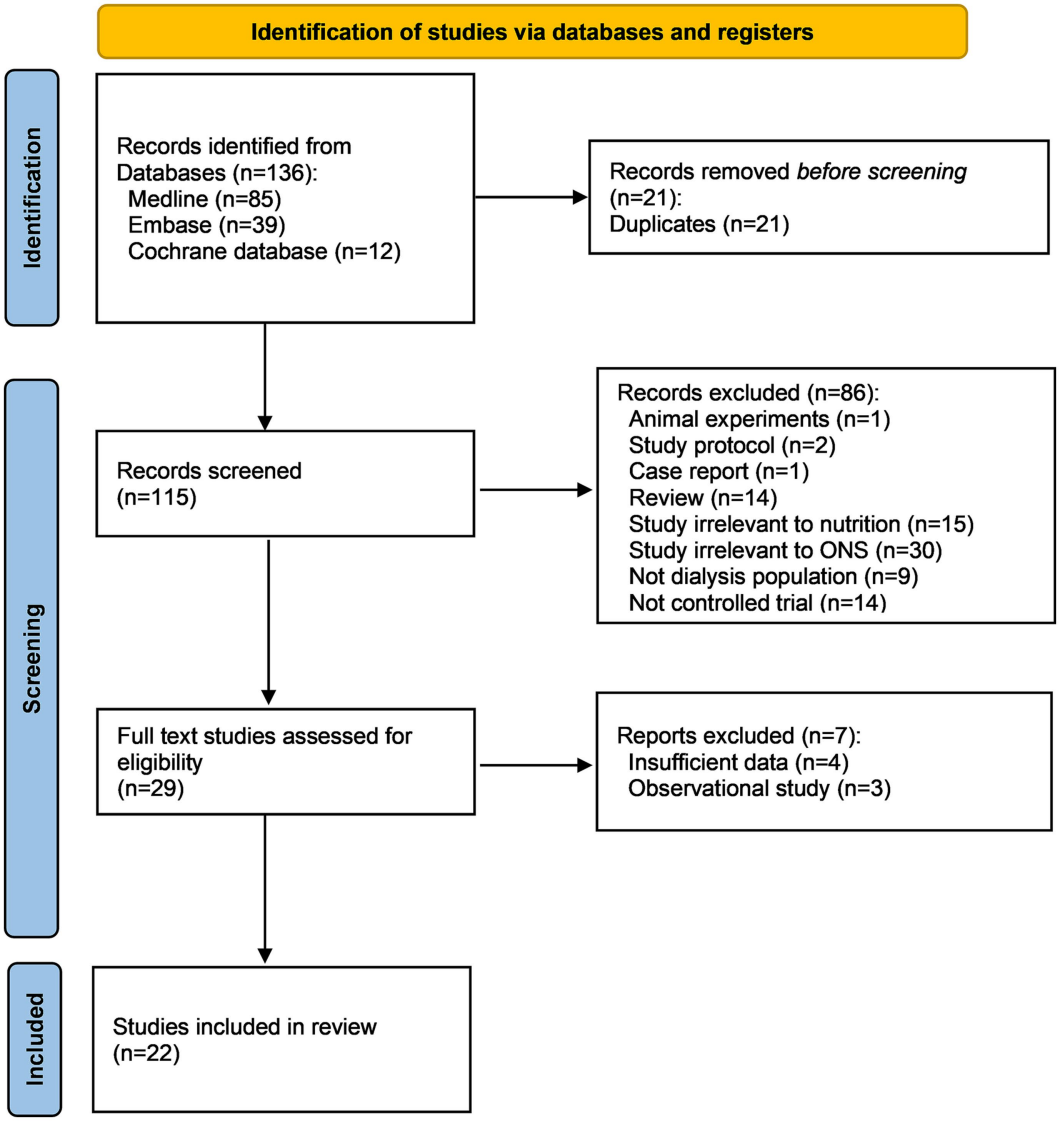
PRISMA flow chart. ONS, oral nutritional supplement.

One hundred thirty-six records were returned from the literature searching. After removing 21 duplicates and 86 articles by screening the titles and abstracts, 29 publications were left for full text review, among which 7 studies were further excluded. Finally, 22 studies encompassing 1,281 patients were included in this systematic review and meta-analysis (12–33) (Figure 1).

### Study characteristics

Nine out of the 22 studies had been conducted in Asia-Pacific region (13, 21, 23, 25–28, 32, 33). There were 4 studied in Middle East (12, 22, 29, 30), 3 studies each in Europe (14, 17, 20) and North America (16, 24, 31), 2 studies in South America (15, 19), and 1 study in Africa (18). Most studies (16/22) had been conducted in hemodialysis (HD) populations, whereas four studies had been investigated in peritoneal dialysis (PD) populations (19, 22, 27, 30). The longest study duration was 6 months. The study population varied greatly from 15 to 240. ALB and BMI were the mostly reported serum indicator and clinical indicator of nutritional status, respectively. The general characteristics of included studies were summarized in Table 1.

**Table 1:** General characteristics of studies included in meta-analysis.

Author/year	Country	Study population	Duration (m)	Total population	ONS treatment		Control treatment		Reported outcomes
					*N*	Regimen	*N*	Regimen	
Afaghi/2016 (12)	Iran	HD; dialyzing ≥6 m, ALB <40 g/L, BMI >18.5 kg/m^2^	6	66	22	ISO-WHEY frequency	22	Routine diet	⑱
					22	BCAA frequency			
Limwannata/2021 (23)	Thailand	HD; dialyzing ≥3 m, ALB <3.8 g/dL, energy intake <25 kcal/kg/day, protein intake <1 g/kg/day	1	80	26	ONCE dialyze (18% protein +42% carbohydrate +40% fat)	24	No intervention	① ④ ⑤ ⑦ ⑩ ⑬ ⑭ ⑮
					30	NEPRO (18% protein +35% carbohydrate +47% Fat)			
Calegari/2011 (15)	Brazil	HD	3	15	9	Oral intradialytic nutritional supplementation	6	Routine nutritional guidance	① ② ③ ④ ⑧ ⑩ ⑬ ⑭ ⑮ ⑱
Bolasco/2011 (14)	Italy	HD; dialyzing ≥6 m, ALB <35 g/L, BMI >20 kg/m^2^, nPCR <1.1 g/kg/d	3	29	15	Oral amino acid supplementation: 4 g bid	14	No other oral supplementation	① ② ⑫ ⑧ ⑩ ⑱
Sahathevan/2018 (27)	Malaysia	PD; dialyzing ≥6 m, ALB <40 g/L, BMI <24 kg/m^2^	6	74	37	WPS (containing 90%–94% whey protein isolate) and dietary counseling	37	Dietary counseling	① ② ③ ④ ⑤ ⑥ ⑦ ⑧ ⑩ ⑬ ⑱
Sharma/2002 (28)	India	HD; dialyzing ≥1 m, ALB <40 g/L, BMI <20 kg/m^2^	1	40	10	CNS formula (500 kcal and 15 g protein)	14	Appropriate dietary counselling	① ② ⑩ ⑬ ⑮
					16	HP formula (500 kcal and 15 g protein)			
Allman/1990 (13)	Australia	HD; dialyzing ≥3 m, BMI <27 kg/m^2^	6	21	9	Glucose polymer (200 kcal)	12	Routine nutritional guidance	① ③ ④ ⑤ ⑥
Eustace/2000 (16)	United States	HD and PD; ALB <38 g/L	3	47	23	Aminess N^®^ tablets (contained 720 mg of amino acids)	24	Placebo	⑩ ⑱
Fouque/2008 (17)	France	HD; dialyzing ≥3 m, ALB <40 g/L, BMI <30 kg/m^2^, nPCR <1.1 g/kg/d	3	86	46	Renilon 7.5^®^ (provided an additional 500 kcal, 18.75 g protein, and 15 mg phosphorus per day)	40	No nutritional supplementation	① ⑩
Gonzalez/2005 (19)	Mexico	PD	6	28	13	ONS (1.3–1.5 g protein/kg/day and 30–35 kcal/kg/day)	15	Routine nutritional guidance	① ② ⑥ ⑩ ⑫ ⑬ ⑭ ⑮ ⑰ ⑱
Hung/2009 (21)	Taiwan	HD; dialyzing ≥6 m	3	41	20	Oral nutritional supplement (contained 16.6 g protein, 22.7 g fat, and 52.8 g carbohydrate and provided 475 kcal)	21	No extra supplementation	① ③ ⑧ ⑩
Imani/2009 (22)	Iran	PD; *p* < 5.5 mg/dL	2	36	18	Soy group (containing 14 g of soy protein and 233 mg of phosphorus)	18	Usual diet	⑬
Rattanasompattikul/2013 (26)	South Korea	HD; ALB <40 g/L	4	43	22	Oral nutritional supplement (Nepro^®^ and AIAO module during hemodialysis sessions)	21	Placebo	⑧ ⑨ ⑩ ⑪ ⑫ ⑬ ⑭ ⑮ ⑯ ⑰
Tabibi/2010 (30)	Iran	PD; *p* < 5.5 mg/dL	2	36	18	Soy group (containing 14 g of soy protein and 233 mg of phosphorus)	18	Usual diet	⑩ ⑪ ⑯ ⑰
Tomayko/2015 (31)	United States	HD; dialyzing ≥3 m, age ≥30	6	38	11	Whey isolate (containing 27 g of soy protein, 151 mg of calcium, 72.6 mg of phosphorus, and 194 mg of potassium)	15	Non caloric placebo powder	③ ⑬ ⑭ ⑮
					12	Soy Isolate (containing 27 g of soy protein, 23 mg of calcium, 244 mg of phosphorus, and 182 mg of potassium)			
Hevilla/2023 (20)	Spain	HD; dialyzing ≥6 m, ALB <35 g/L, BMI <23 kg/m^2^	6	31	20	Oral nutritional supplement (every 100 mL includes 8.97 g of protein, 8.7 g of fat, and 1.2 g of sugar, and provided 200 kcal)	11	Individualized dietary recommendations	⑤ ⑧ ⑨ ⑩ ⑪ ⑫ ⑬ ⑭ ⑮ ⑯ ⑰
Qin/2022 (25)	China	HD; dialyzing ≥3 m, age ≥18, diagnosed as PEW	2	37	19	Oral nutritional supplement (Fresubin^®^, one bottle of OES (120 mL) contains 600 kcal of energy, 4.0 g of carbohydrates, and 53.8 g of lipids)	18	Dietary recommendations	① ⑤ ⑦ ⑩ ⑫ ⑬ ⑭ ⑮ ⑯ ⑰
Wen/2022 (32)	China	HD; dialyzing ≥3 m, age ≥18	6	92	49	Oral nonprotein calorie (each serving (90 g) contained 140 kcal of energy, 5.4 g of fat, and 22.5 g of carbohydrate)	43	Dietary counselling	① ⑤ ⑥ ⑧ ⑨ ⑩ ⑪ ⑫ ⑬ ⑭ ⑯ ⑰ ⑱
Yang/2021 (33)	China	HD; dialyzing ≥3 m, ages ≥18	3	240	120	Oral supplement (Fresubin^®^, provides 97% of energy)	120	No nutritional supplementation	① ⑥ ⑩ ⑪ ⑫
Gharib/2023 (18)	Egypt	HD; dialyzing ≥6 m, diagnosed as PEW, ALB <35 g/L, pre-ALB <20 mg/dL	3	60	30	Oral nutritional supplement (every 100 g includes 26.7 g of protein, 12.08 g of fat, and 52.58 g of carbohydrate and provided 423 kcal)	30	Usual diet	① ② ④ ⑧ ⑩ ⑪ ⑫ ⑬ ⑭ ⑮ ⑰
Sohrabi/2016 (29)	Iran	HD; age: 17–65	2	92	23	Fermented vitamin E2 fortified whey beverage (15 g of whey protein concentrate 1,600 IU of vitamin E)	23	No intervention	① ⑦ ⑧ ⑨ ⑩ ⑭ ⑮ ⑯ ⑰
					23	Fermented whey beverage (15 g of whey protein concentrate)			
					23	Vitamin E (600 IU)			
Moretti/2009 (24)	United States	HD and PD; dialyzing ≥3 m	6	49	31	HD: oral 15 g liquid hydrolyzed collagen protein supplement tiw; PD: oral 15 g of protein qd	18	No supplementation received	② ⑩

Reported outcomes: ① BMI, body mass index; ② nPCR, normalized protein catabolic rate; ③ fat mass; ④ lean mass; ⑤ MAC, mid arm circumference; ⑥ MAMC, mid arm muscle circumference; ⑦ MIS, malnutrition inflammation score; ⑧ CRP, C-reaction protein; ⑨ IL-6, interleukin-6; ⑩ ALB, albumin; ⑪ pre-ALB, pre-albumin; ⑫ HGB, hemoglobin; ⑬ P, phosphorus; ⑭ Ca, calcium; ⑮ K, potassium; ⑯ TC, total cholesterol; ⑰ TG, triglyceride; ⑱ Kt/V. HD, hemodialysis; m, month; PD, peritoneal dialysis; PEW, protein energy wasting; tiw, three times per week.

### Effects of ONS on laboratory indicators

**Figure 2:**
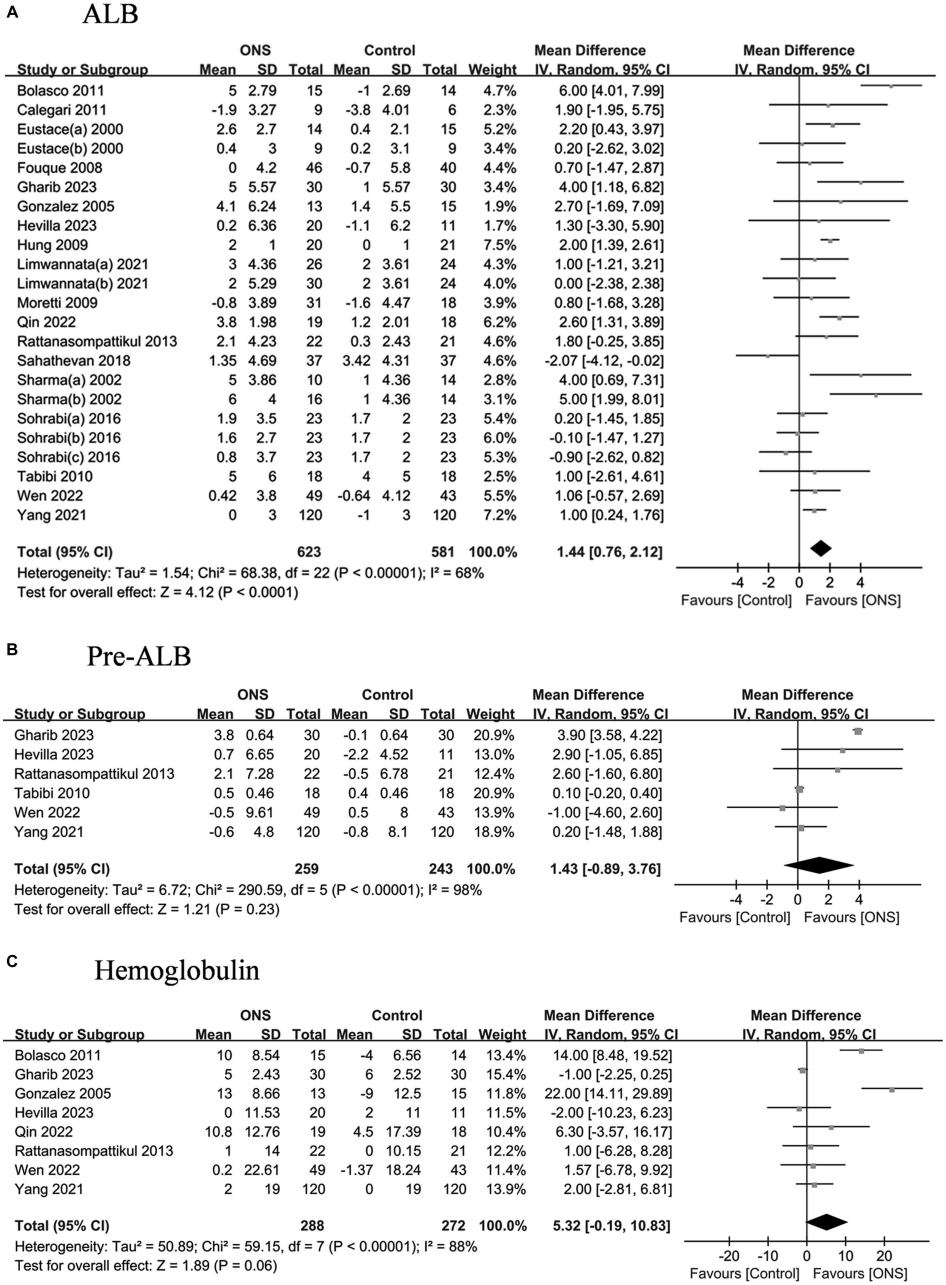
Pooled analysis of the effects of ONS treatment on laboratory indicators: **(A)** Albumin; **(B)** Pre-albumin; **(C)** Hemoglobulin. Abbreviations: ALB, albumin; pre-ALB, pre-albumin.

The pooled analysis indicated that ONS treatments significantly improved the serum ALB by 1.44 g/L (95% CI: 0.76, 2.57, *p* < 0.001) yet with high heterogeneity (*I*^2^ = 68.0%, *p* < 0.001) (Figure 2); however, the ONS treatments did not show significant effects on the levels on other laboratory indicators, including pre-ALB, hemoglobulin, electrolytes (Supplementary Figure S1), or lipid (Supplementary Figure S2).

### Effects of ONS on anthropometric and body composition analysis measures

**Figure 3:**
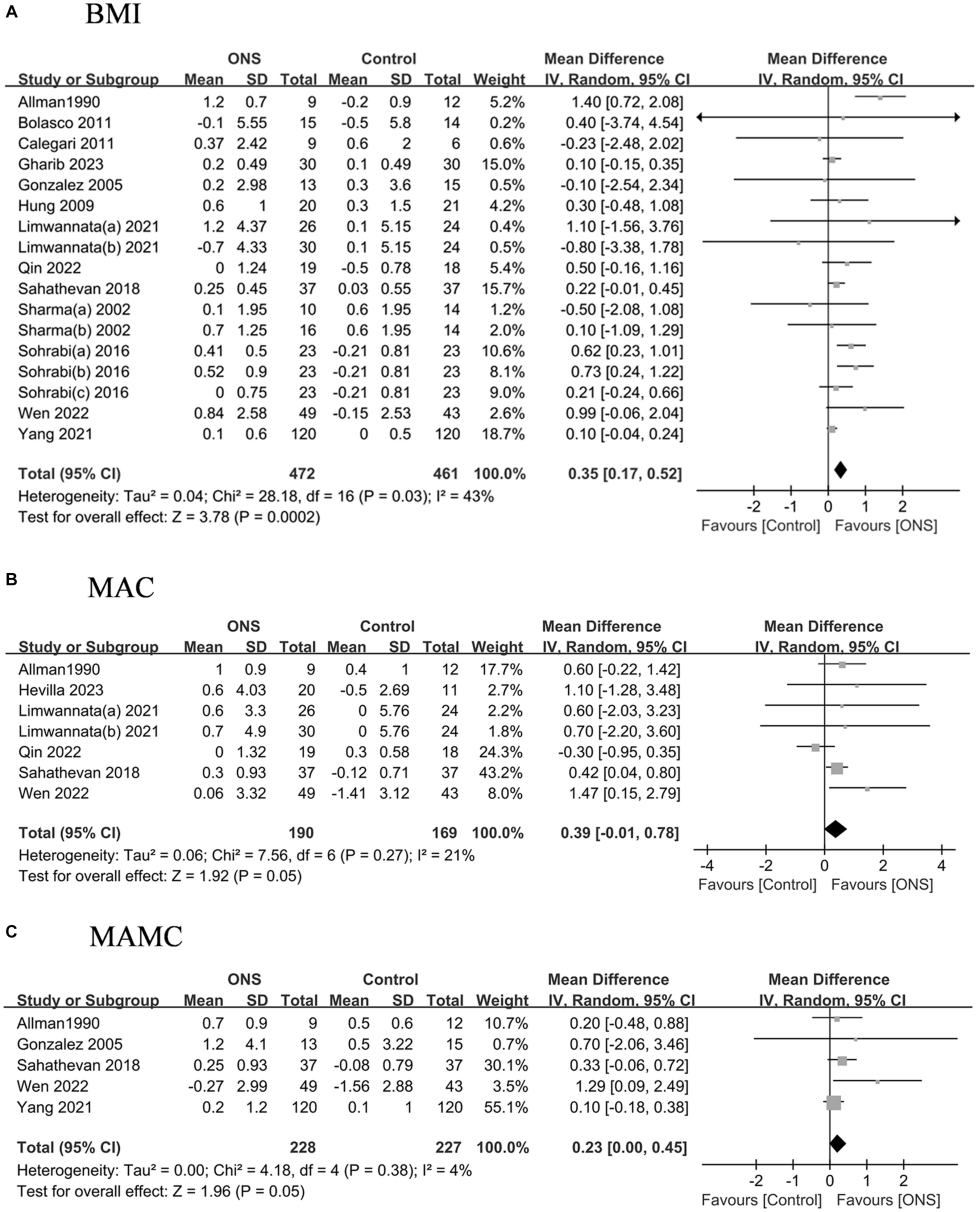
Pooled analysis of the effects of ONS treatment on anthropometric measures: **(A)** BMI; **(B)** MAC; **(C)** MAMC. Abbreviations: BMI, body mass index; MAC, mid arm circumference; MAMC, mid arm muscle circumference.

**Figure 4:**
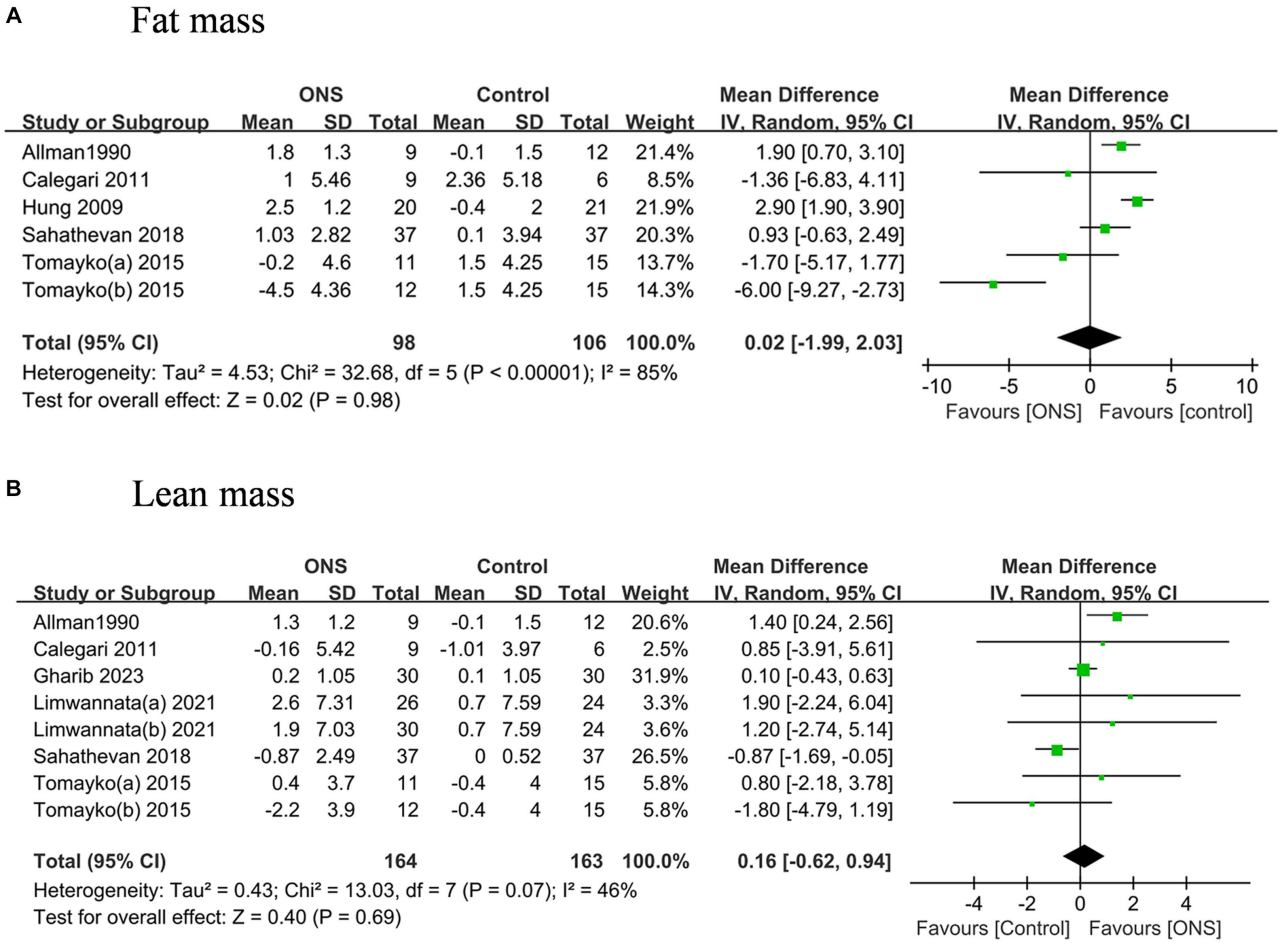
Pooled analysis of the effects of ONS treatment on body composition analysis measures: **(A)** Fat mass; **(B)** Lean mass.

The pooled analysis indicated the ONS treatments significantly improved the BMI by 0.35 kg/m^2^ (95% CI: 0.17, 0.52, *p* = 0.002) with moderate heterogeneity (*I*^2^ = 43%, *p* = 0.03) (Figure 3). The changes of MAC and MAMC values after the ONS treatments did not differ from those after the control treatments. The pooled analysis indicated the ONS treatments did not change the fat mass or the lean mass derived from body composition analysis (Figure 4).

### Effects of ONS on nutritional indices

**Figure 5:**
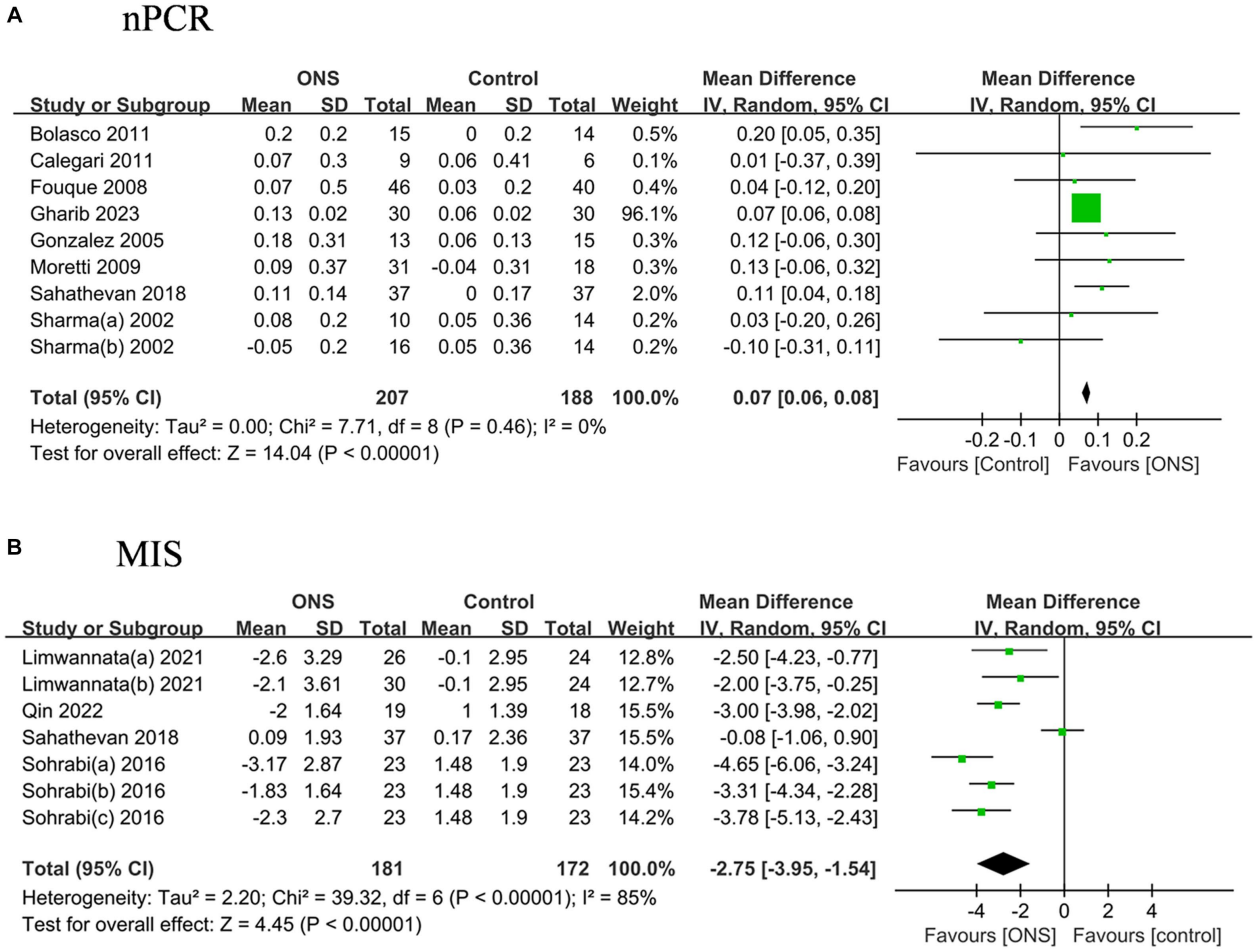
Pooled analysis of the effects of ONS treatment on nutritional indices: **(A)** nPCR; **(B)** MIS. Abbreviations: nPCR, normalized protein catabolic rate; MIS, malnutration inflammation score.

The pooled nPCR was significantly improved after the ONS treatments by 0.07 g/(kg d) (95% CI: 0.05, 0.10, *p* < 0.001) with low heterogeneity (*I*^2^ = 0%, *p* = 0.46) (Figure 5). Similarly, the pooled MIS decreased significant after the ONS treatments by 2.75 (95% CI: −3.95, −1.54, *p* < 0.001) with high heterogeneity (*I*^2^ = 83.5%, *p* < 0.001).

### Effects of ONS on dialysis adequacy and inflammation indicators

**Figure 6:**
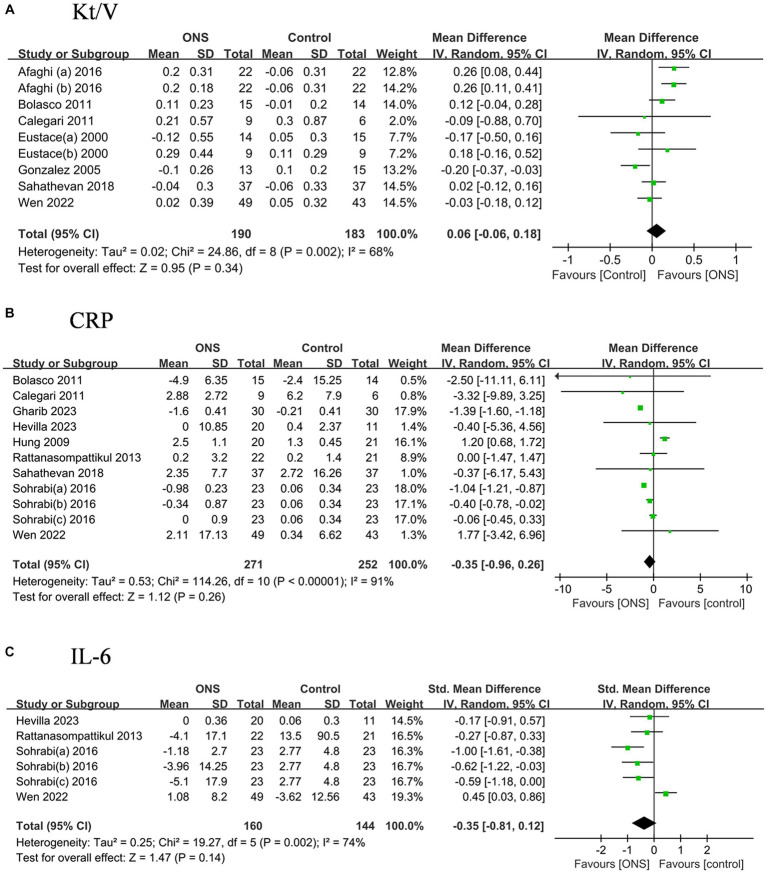
Pooled analysis of the effects of ONS treatment on dialysis adequacy and systemic inflammation indicators: **(A)** Kt/V; **(B)** CRP; **(C)** IL-6. Abbreviations: CRP, C-reaction protein; IL-6, Interleukin-6.

The dialysis adequacy reflected by Kt/V did not show significant improvement after the ONS treatments compared to the control treatments, nor the levels of CRP and IL-6 (Figure 6).

### Subgroup analyses

To further explore the heterogeneities of four outcomes that exhibited significant improvement after ONS treatment, namely ALB, BMI, nPCR, and MIS, subgroup analyses based on follow up duration and dialysis mode were conducted. The results indicated the improvement of the nPCR insignificantly increased with longer study duration, whereas the improvement of ALB, BMI, and MIS after ONS treatments was only significant in patients receiving treatments less than 3 months (Supplementary Figure S3). Similarly, the improvement of ALB, BMI, and MIS after ONS treatments was only significant in HD populations, whereas the improvement of the nPCR was significant both in HD and PD populations (Supplementary Figure S4).

### Critical appraisal

Based on the Cochrane criteria, none of the included studies had low risk of bias (Supplementary Figure S5). Fifteen studies were rated as having high risk of bias. The mostly common reason for high risk of bias was the lack of double blindness in the study design in all except for three studies.

### Publication bias

Visual inspection of the funnel plot revealed relative symmetry, arguing against the presence of publication bias (Supplementary Figure S6). This finding was supported by the results of Egger test (*p* = 0.973). There were 5 studies outside the plot, indicating the presence of heterogeneity.

## Discussion

The results of this systematic review and meta-analysis suggest that ONS treatment helps to improve the nutritional status of dialysis dependent patients by exhibiting a positive impact on BMI, serum ALB, nPCR, and MIS when compared to control treatments. However, no significant differences were observed in relation to the other outcomes examined. It is important to note that a majority of the studies included in our analysis were deemed to have a high risk of bias.

Oral supplementation of energy, protein, or lipid has been shown to be advantageous for patients dependent on dialysis (8). To comprehensively cover currently available nutritional treatment options, this study considered various forms of supplements, from commercially available lipid fluid to self-formulated supplements, from a mixture of multiple nutrients to a single nutrient such as vitamin E. Taken together, our findings revealed that ONS treatments resulted in significant improvements in BMI, serum ALB, and nPCR levels, without affecting electrolyte levels that are susceptible to dietary influences such as phosphorus, which align with existing literature (8). It should be noted the wide variations in the types of ONS included in this meta-analysis might be a source of the observed heterogeneities. Other potential sources of the heterogeneities include the nutritional status in the inclusion criteria, dialysis vintage of the population, duration of treatment, and dialysis mode. The results of subgroup analyses indicated the improvement of ALB, BMI, and MIS after ONS treatments was significant in HD populations and in patients receiving short-term treatment, and the improvement in nPCR was not affected by dialysis mode or treatment duration, suggesting the benefits of ONS might be more easily observed in HD patients.

This study is the first to report evidence of improved MIS following ONS treatments through a meta-analysis of results from RCTs. The MIS serves as a comprehensive evaluation of nutritional status of dialysis patients from four dimensions (4, 5). The MIS reflects the risk of malnutrition and has been reported to significantly correlate with morbidity and mortality in maintenance dialysis patients (3, 34, 35); therefore, the improvement in the MIS is of important clinical relevance in this population. The improvement of nutritional status and microinflammatory environment inside the body can augment patients’ resistance to infections, mitigate the advancement of arterial diseases, optimize nutrients utilization, and ultimately resulting in an improved long-term prognosis (3, 5).

The absence of discernible advantages of ONS treatments compared to control treatments across all various outcomes investigated may be attributed to the multifaceted nature of nutritional status, which is influenced by factors far beyond oral intake alone. Even oral intake itself is significantly influenced by various factors, including cultural practices, personal habits, family habits, and food availability, among which ONS represents only one therapeutic element. The control treatments in the majority of the included studies employed routine nutritional guidance and diet counseling. Additionally, patient education is frequently and causally provided by healthcare professionals during dialysis sessions. These interventions have been shown to enhance nutritional status (36–38), but their implementation in real-world settings poses challenges in terms of standardization, thereby introducing confounding factors in clinical trials. Consequently, this lack of standardization may account for the lack of significant differences observed between ONS treatments and control treatments in the present meta-analysis.

The potential influence of study duration on the impact of ONS on nutritional indices should also be taken into account. The outcomes on which ONS exhibited beneficial effects in this study, namely BMI, serum ALB, nPCR, and MIS, were all short-term outcomes. These measures provide rapid indications of changes in nutritional status within the body. Conversely, longer treatment durations may be necessary to observe any changes in long-term outcomes, such as MAC and MAMC derived from anthropometry. In addition, the adherence to ONS is an important component in the long-term management of renal failure patients, which might be enhanced by early onset of beneficial effects and professional patient education. Another important consideration of ONS is the cost, particularly in regions where commercially available ONS agents are relatively expensive and not covered by social medical insurance. Long-term use of ONS needs supportive evidence from cost-effective analysis.

The current study benefited from its comprehensive evaluation encompassing multiple facets of malnutrition in dialysis patients, including laboratory indicators, anthropometric measures, dialysis adequacy, diet evaluation, body composition analysis measures, and systemic inflammation indicators. There are several limitations that should be acknowledged. Firstly, a majority of the studies included in the analysis (13 out of 21) had a sample size of less than 50. Secondly, 14 out of the 21 studies were determined to have a high risk of bias, which hinders the ability to draw strong and reliable conclusions. Thirdly, the control treatments utilized in the included studies varied significantly, potentially introducing interference in the comparison. Lastly, due to a lack of reporting, we were unable to assess the impact of ONS on long-term outcomes, as the longest study duration was limited to 6 months.

## Conclusion

This systematic review and meta-analysis suggest that ONS treatments help to improve nutritional status among dialysis dependent patients by exhibiting a positive impact on BMI, serum ALB, nPCR, and MIS when compared to control treatments. More evidence is needed from future investigations with longer study duration and standardized procedures to support long-term use of ONS in this population.

## Data availability statement

The original contributions presented in the study are included in the article/Supplementary material, further inquiries can be directed to the corresponding author.

## Author contributions

SoR: Conceptualization, Data curation, Formal analysis, Methodology, Software, Writing – original draft. XY: Data curation, Formal analysis, Writing – original draft. ShR: Formal analysis, Writing – review & editing. YF: Formal analysis, Writing – review & editing, Conceptualization, Data curation, Methodology, Project administration, Supervision.
