# Supplementary material for: Oral nutritional supplement helps to improve nutritional status of dialysis dependent patients: a systematic review and meta-analysis of randomized controlled trials
Source: Front Nutr. 2023 Nov 23;10:1294064. doi: 10.3389/fnut.2023.1294064 (PMC10702223; doi:10.3389/fnut.2023.1294064)

**Supplementary Materials**

Supplementary Table 1. Search terms used in literature searching.

Supplementary Figure 1. Pooled analysis of the effects of ONS treatment on electrolytes examinations.

Supplementary Figure 2. Pooled analysis of the effects of ONS treatment on lipid examinations.

Supplementary Figure 3. Subgroup analysis based on the study duration of four outcomes that showed significant improvement after ONS treatments.

Supplementary Figure 4. Subgroup analysis based on the dialysis of four outcomes that showed significant improvement after ONS treatments.

Supplementary Figure 5. Critical appraisal based on the Cochrane criteria.

Supplementary Figure 6. Funnel plot for publication bias evaluation.

Supplementary Table 1. Search terms used in literature searching.

| Database | Search terms |
| --- | --- |
| Embase via Ovid | 1. random*.mp.  2. randomized controlled trial.mp.  3. clinical trial.mp.  4. control trial.mp.  5. 1 OR 2 OR 3 OR 4  6. chronic kidney disease.mp.  7. dialysis.mp.  8. hemodialysis.mp.  9. peritoneal dialysis.mp.  10. 6 OR 7 OR 8 OR 9  11. nutrition.mp.  12. oral supplement.mp.  13. ONS.mp.  14. nutrient*.mp.  15. macronutrients.mp.  16. calorie supplement.mp.  17. energy supplement.mp.  18. protein supplement.mp.  19. amino acid supplement.mp.  20. 11 OR 12 OR 13 OR 14 OR 15 OR 16 OR 17 OR 18 OR 19  21. 5 AND 10 AND 20 |
| Medline via Pubmed | (random* OR (Randomized controlled trial) OR (Clinical trial) OR (Control trial)) AND ((chronic kidney disease) OR dialysis OR Hemodialysis OR (Peritoneal dialysis)) AND (nutrition OR (oral supplement) OR ONS OR nutrient OR macronutrients OR (calorie supplement) OR (energy supplement) OR (protein supplement) OR (amino acid supplement)) |
| Cochrane Central via Ovid | 1. random*.mp.  2. randomized controlled trial.mp.  3. clinical trial.mp.  4. control trial.mp.  5. 1 OR 2 OR 3 OR 4  6. chronic kidney disease.mp.  7. dialysis.mp.  8. hemodialysis.mp.  9. peritoneal dialysis.mp.  10. 6 OR 7 OR 8 OR 9  11. nutrition.mp.  12. oral supplement.mp.  13. ONS.mp.  14. nutrient*.mp.  15. macronutrients.mp.  16. calorie supplement.mp.  17. energy supplement.mp.  18. protein supplement.mp.  19. amino acid supplement.mp.  20. 11 OR 12 OR 13 OR 14 OR 15 OR 16 OR 17 OR 18 OR 19  21. 5 AND 10 AND 20 |

Supplementary Figure 1. Pooled analysis of the effects of ONS treatment on electrolytes examinations.


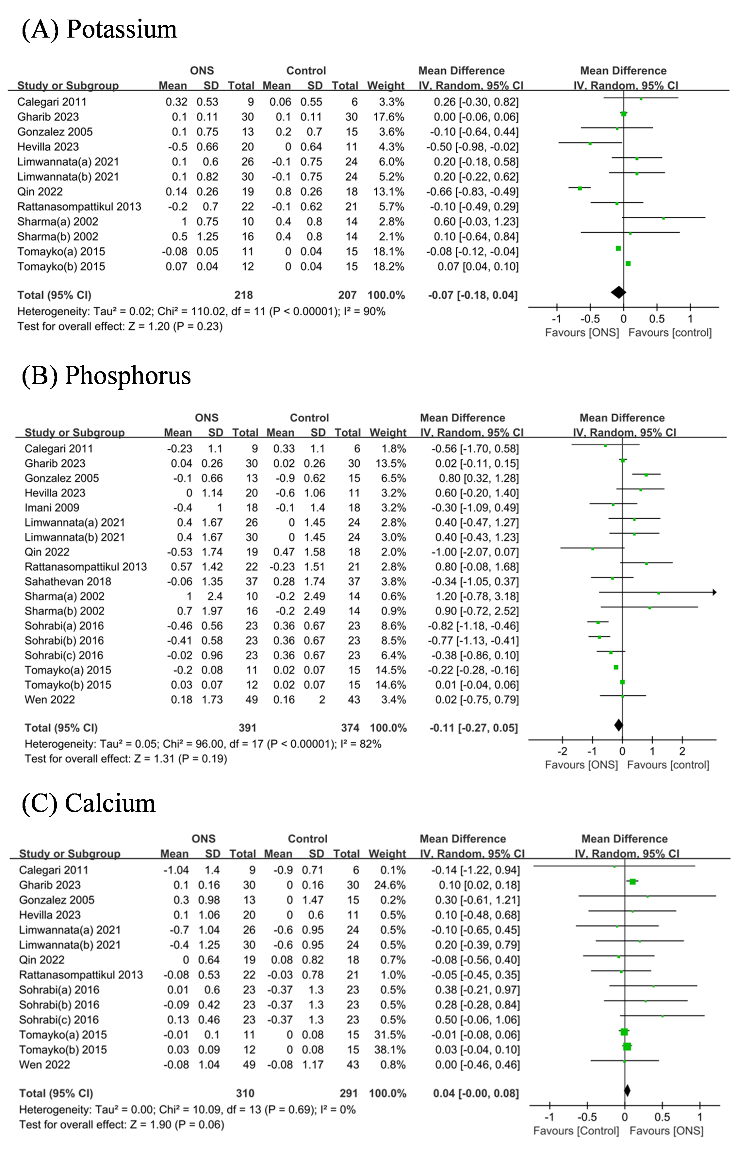


Supplementary Figure 2. Pooled analysis of the effects of ONS treatment on lipid examinations.


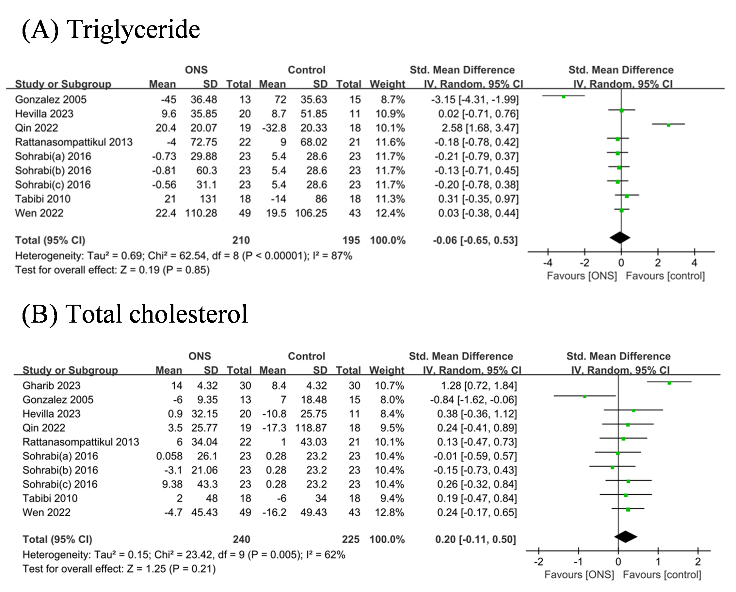


Supplementary Figure 3. Subgroup analysis based on the study duration of four outcomes that showed significant improvement after ONS treatments.


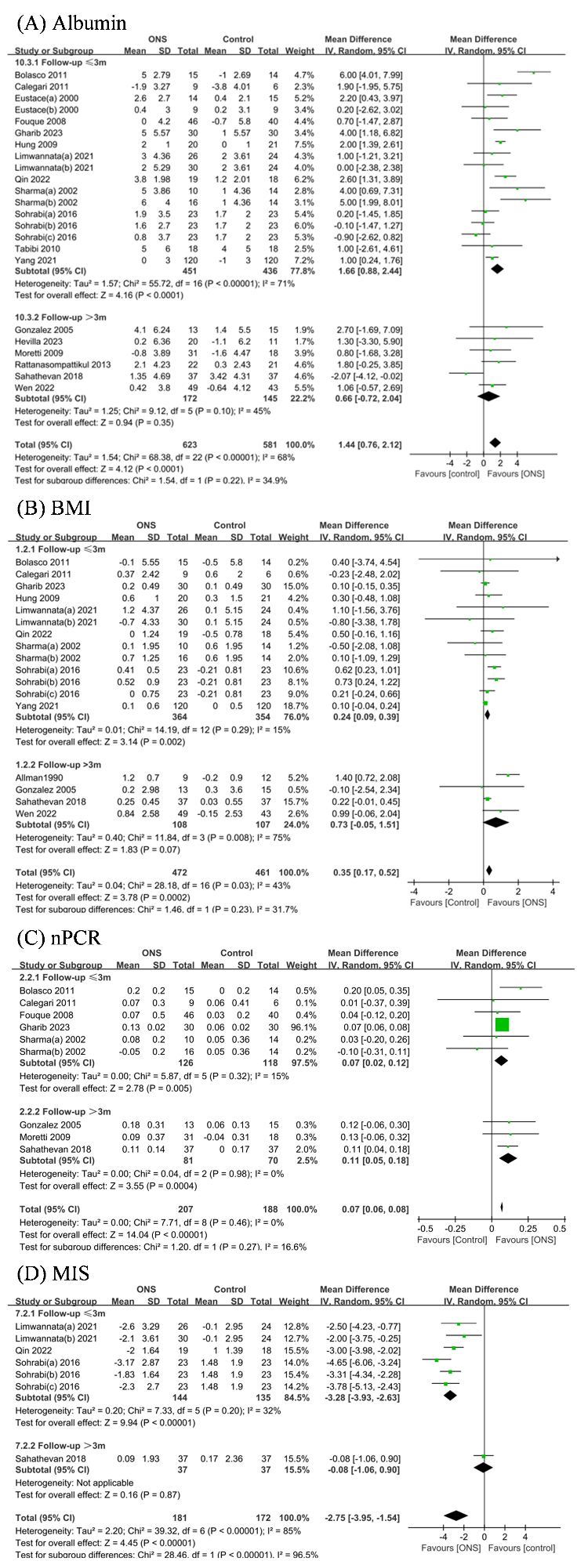


Supplementary Figure 4. Subgroup analysis based on the dialysis of four outcomes that showed significant improvement after ONS treatments.


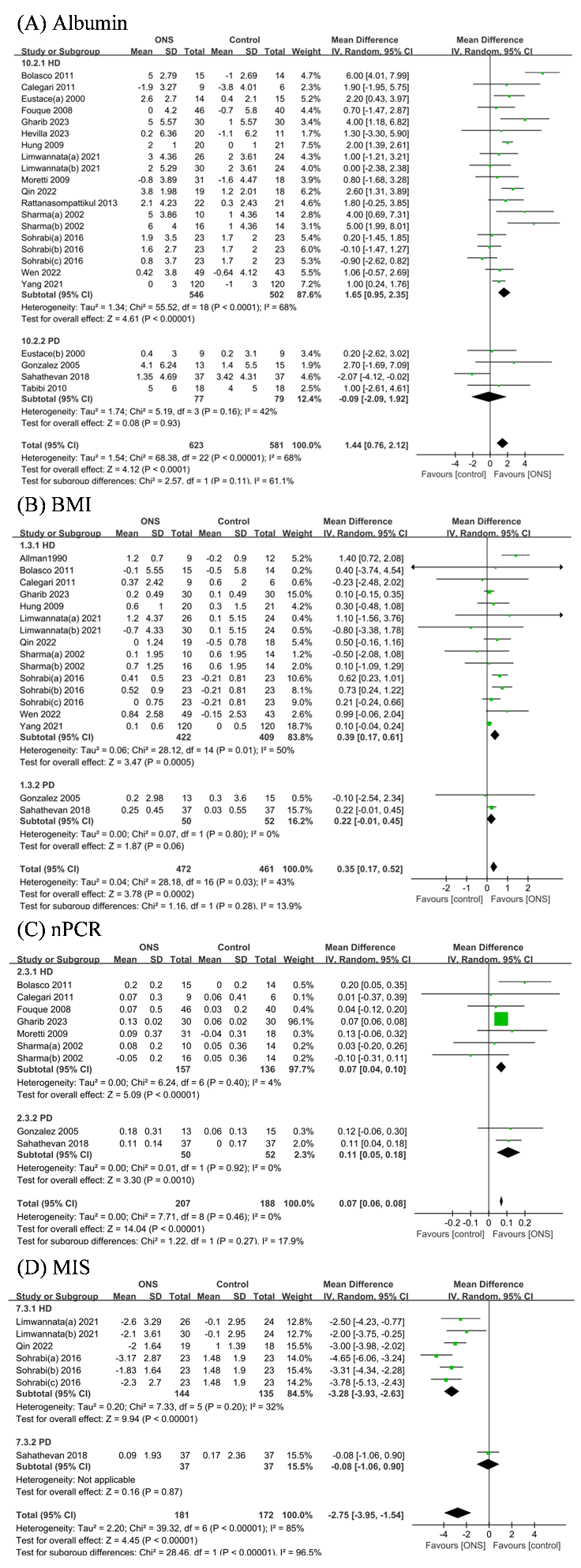


Supplementary Figure 5. Critical appraisal based on the Cochrane criteria.


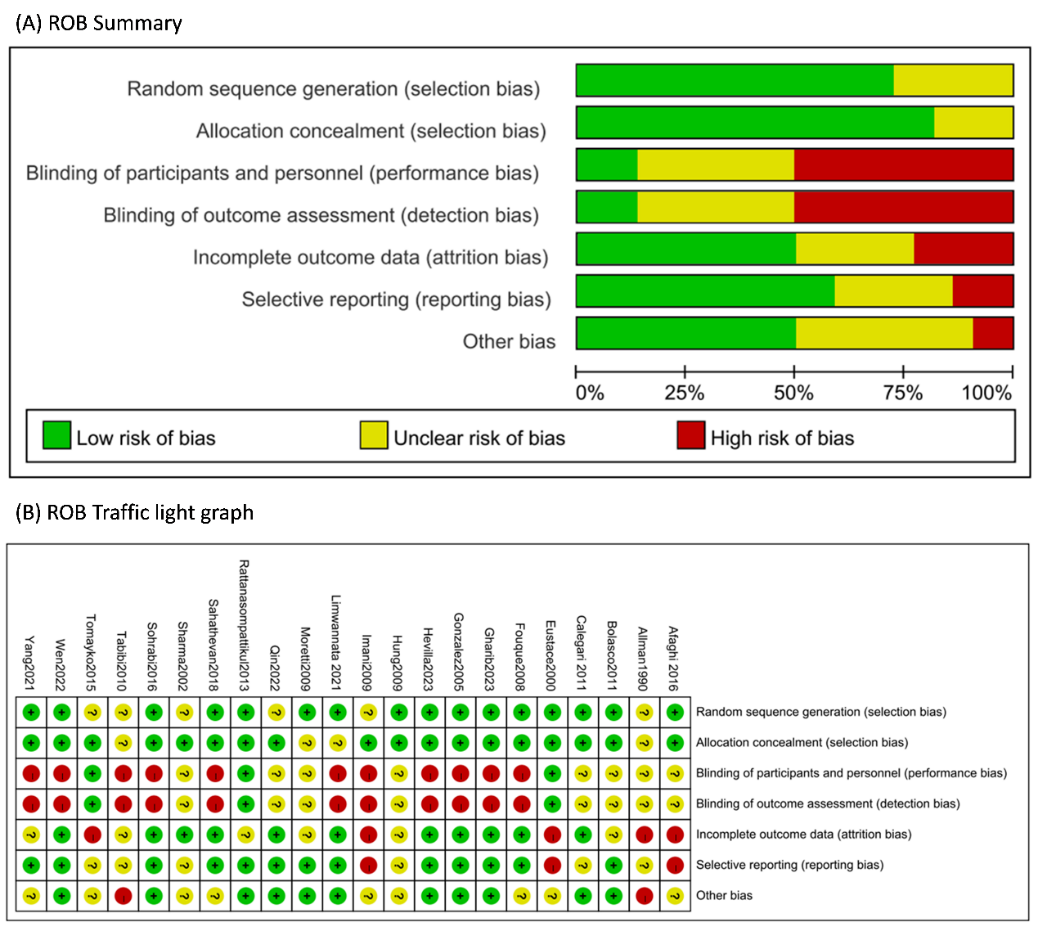


Supplementary Figure 6. Funnel plot for publication bias evaluation.


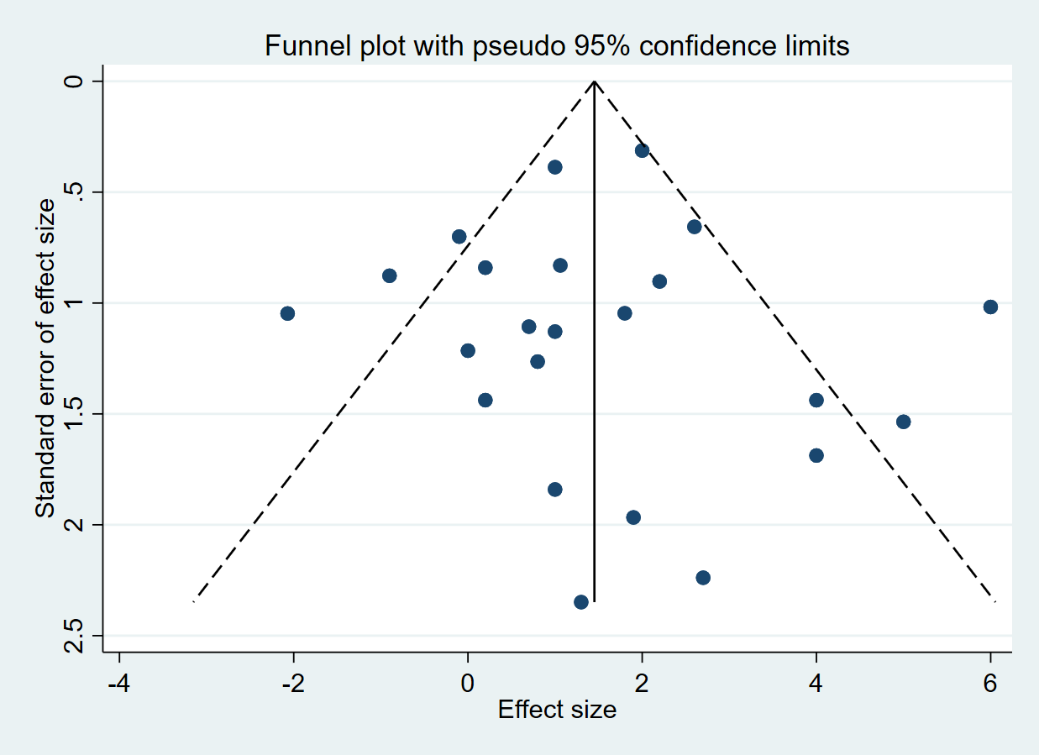

Supplement: Supplementary file 1 [file Data_Sheet_1.docx]
